# Reactivation of Disseminated Histoplasmosis With Central Nervous System Involvement Following a Primary Gastrointestinal Histoplasmosis Infection: A Case Report

**DOI:** 10.7759/cureus.28553

**Published:** 2022-08-29

**Authors:** Margaret McGrath, Rob Nguyen, Evgeniya Tyrtova, Ali C Ravanpay

**Affiliations:** 1 Neurological Surgery, University of Washington, Seattle, USA; 2 School of Medicine, University of Washington, Seattle, USA

**Keywords:** ring-enhancing lesions, central nervous system, gastrointestinal histoplasmosis, case report, cns histoplasmosis

## Abstract

A 78-year-old white male with chronic pancytopenia presented with acute transient aphasia and dysarthria. He had a National Institutes of Health Stroke Scale (NIHSS) of zero. Physical examination revealed slight aphasia with mild dysarthria. Brain magnetic resonance imaging (MRI) revealed nine ring-enhancing lesions in the left precentral gyrus with significant vasogenic edema. Lung computed tomography (CT) showed no evidence of pulmonary nodules. The serology of blood and urine for infectious organisms was negative. Four weeks later, the patient was re-admitted with worsening dysarthria and right upper extremity weakness. Repeat head MRI showed a slight increase in the size of the multiple supratentorial ring-enhancing lesions. The magnetic resonance spectroscopy (MRS) findings of the evaluated lesion suggested a fungal etiology. Empiric amphotericin B treatment was initiated, which mitigated central nervous system (CNS) ring-enhancing lesions and resolved the patient’s neurological deficits. Early empiric medical treatment of CNS histoplasmosis should be considered in the setting of multiple CNS ring-enhancing lesions and a positive history of histoplasmosis infection, despite negative serological studies.

## Introduction

Histoplasmosis is an infection caused by the dimorphic fungus *Histoplasma capsulatum* and is one of the most common fungal respiratory infections globally, especially in immunocompromised hosts. In the United States (US), it is endemic to central and southeastern states, particularly in the lower Ohio Valley and Mississippi River. An estimated 40 million people in the US are infected with Histoplasma [1]. *H. capsulatum* is a dimorphic fungus, taking on mycelia and yeast forms in cold and hot temperatures, respectively. This fungus feeds on nitrogen-rich soil, particularly those containing bird or bat droppings. When deposited in soil, *H. capsulatum* survives in its mycelia form. With soil disturbance, *H. capsulatum* can become airborne and inhaled into the lungs. Due to higher temperatures in the lungs compared with the temperature of the soil, *H. capsulatum* takes on its yeast form and becomes phagocytosed by pulmonary macrophages, where it avoids cellular-mediated immunity [1].

Histoplasmosis can progress to be disseminated in patients with acute infection. In patients with a previous history of infection, there is a risk of future re-infection, primarily among those with impaired immunity [2,3]. Central nervous system (CNS) histoplasmosis is a rare manifestation seen in 5% to 10% of patients with disseminated histoplasmosis [4]. The risk of developing CNS histoplasmosis is highest in immunocompromised individuals, although a few isolated cases have been identified in immunocompetent patients [5-8]. The most sensitive method for diagnosis of CNS histoplasmosis is the detection of the antibody and antigen in the cerebrospinal fluid (CSF). Once diagnosed, the recommended guideline for the treatment of CNS histoplasmosis is amphotericin B, followed by itraconazole for at least one year. Upon review of the literature via a PubMed search for "reactivated disseminated histoplasmosis in the CNS," one prior report of reactivated disseminated histoplasmosis in the CNS was identified [3].

We present a case of reactivation of CNS histoplasmosis following a primary diagnosis of seronegative gastrointestinal histoplasmosis in an immunocompetent patient. To the best of our knowledge, there are very few reports of reactivated disseminated histoplasmosis presenting as CNS histoplasmosis [3], and none with corresponding imaging findings.

## Case presentation

A 78-year-old white male, medically complex but immunocompetent, presented with transient aphasia and acute dysarthria. The patient’s past medical history was significant for asymptomatic seronegative gastrointestinal (GI)-disseminated histoplasmosis, diagnosed during a routine colonoscopy three years prior. After appropriate treatment at that time, he showed no evidence of the dissemination of the disease. In addition, he had chronic pancytopenia, adnominal aortic aneurysm repair, adrenal insufficiency, hyperlipidemia, stage 3 chronic kidney disease, hypertension, hypothyroidism, and significant peripheral vascular disease. The patient was on appropriate medications for his respective comorbidities. His social history was notable for minimal alcohol use, a remote smoking history, and no illicit or intravenous drug use. The patient endorsed living in Pittsburgh, Pennsylvania, United States, for a short time, but otherwise had no known travel history to Histoplasma endemic areas.

**Figure 1:**
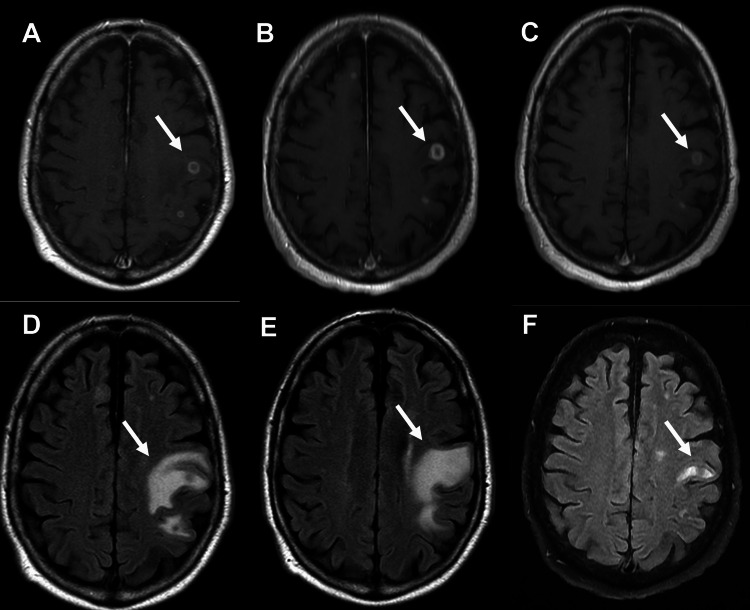
Imaging showing lesions and edema in the left precentral gyrus before and after treatment initiation. (A) Initial axial T1 with contrast MRI showing multifocal ring enhancing lesions with associated vasogenic edema with the largest lesion shown by the white arrow. (B) Axial T1 with contrast MRI showing multifocal ring enhancing lesions with associated vasogenic edema with progression of size/edema on imaging. This image is four weeks after presentation without any treatment. Arrow indicates the largest lesion. (C) Axial T1 with contrast MRI showing multifocal ring enhancing lesions with associated vasogenic edema, with reduction in size and edema, four months after treatment initiation. Arrow indicates the largest lesion. (D) Axial T1 flair on initial presentation, showing vasogenic edema as indicated by the arrow. (E) Axial T1 flair showing progression of vasogenic edema, four weeks after presentation without any treatment. Arrow indicates edema from largest lesion. (F) Axial T1 flair four months after starting treatment, showing reduction in vasogenic edema. Arrow indicates reduced edema from the largest lesion.

On physical examination, the patient was afebrile. His neurologic exam was notable for stable dysarthria but otherwise neurologically intact on initial presentation. A complete blood count (CBC) showed leukocytes (white blood cells, WBC) 3.43 K/μL, red blood cells (RBC) 4.04 Mil/μL, lymphocytes 100/μL, and platelets 101 K/μL. Lung computed tomography (CT) was unremarkable. An abdominal CT revealed splenomegaly. The patient underwent magnetic resonance angiography (MRA) and magnetic resonance imaging (MRI) of the brain and neck, which revealed multiple ring-enhancing lesions in the left precentral gyrus, with significant vasogenic edema (Figure 1). 

Infectious disease specialists were consulted and explored infectious versus malignant etiologies. Given the patient’s history, urine and serum Histoplasma antigen were tested and found to be negative. Blood cultures for Histoplasma were also negative. Tuberculosis (TB) testing (QuantiFERON-TB Gold), Toxoplasma serology, and HIV antibody screening were all negative, with the latter test showing P24 antigen positivity. A positive P24 antigen test could indicate a positive HIV infection. However, given the negative HIV antibody results, in this situation, infectious disease was not reflective of an active HIV infection but rather was a false positive result. For further diagnostic clarity, a lumbar puncture (LP) was considered, but because the patient was on dual anti-platelet therapy (DAPT) and anticoagulation, the decision was made to not pursue an LP at that time. The patient was subsequently discharged, and magnetic resonance spectroscopy (MRS) was recommended for follow-up.

**Figure 2:**
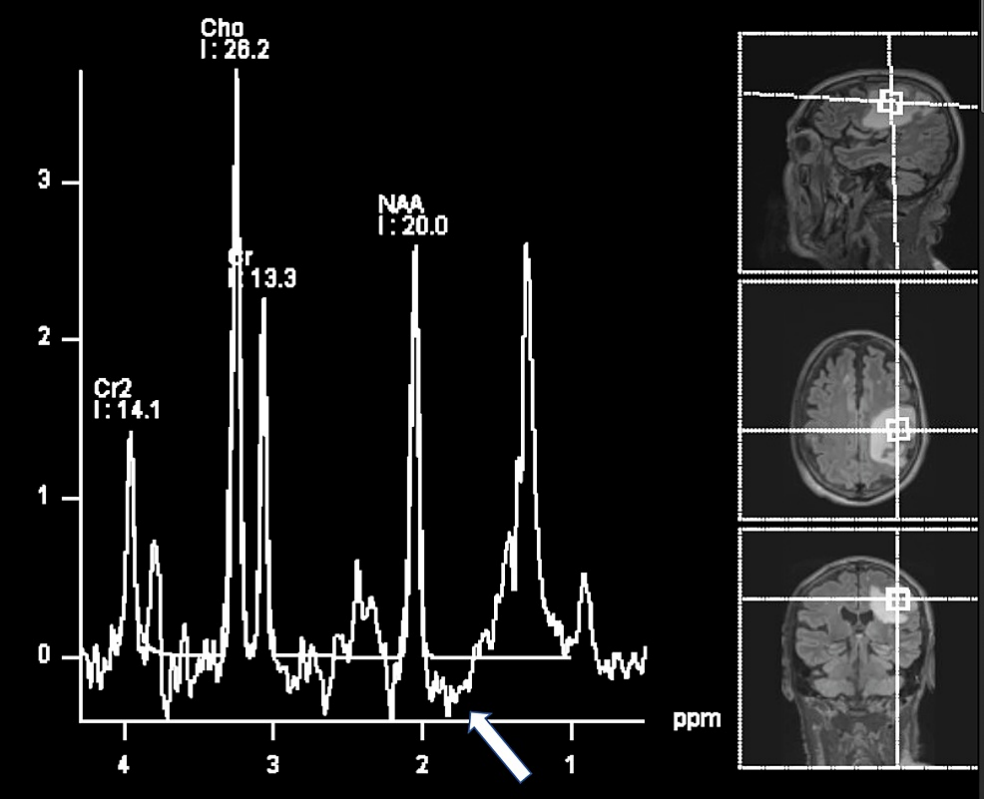
Magnetic resonance spectroscopy findings. MRS imaging showing increased peak at the expected region of lipid/lactate (arrow) but without inversion on medium TE sequences, and with possible contributions from metabolites such as alanine.

The patient returned weeks later with a worsening of his symptoms. Upon physical examination, the patient remained afebrile. He had slight dysarthria and a new right arm weakness. On motor examination, the patient had a notable right pronator drift and 4+/5 strength on the manual assessment of muscle strength grading scale throughout his right upper extremity. He had full strength 5/5 muscle strength in his left upper and lower extremities and his right lower extremity. The rest of the patient’s neurological exam was normal. A CBC showed WBCs 2.22 K/μL (with 38% neutrophils and 85% mature), RBCs at 3.44 Mil/μL, and platelets at 87 K/μL. Repeat Histoplasma urine and serum antigen detection tests were negative. A positron emission topography (PET) scan showed no evidence of metastasis. Repeat head MRI showed a slight increase in size of the multiple supratentorial ring-enhancing lesions since his initial presentation (Figure 1). MRS findings demonstrated an increased peak at the expected region of lipid/lactate but without inversion on medium time to echo (TE) sequences and with possible contributions from metabolites such as alanine (Figure 2).

The overall MRS findings of the evaluated lesion favored an infectious, fungal etiology based on the corresponding lipid and lactate peaks being highly suggestive [9]. Our infectious disease specialists did not recommend starting empiric anti-microbial treatment because the patient was seronegative for all infectious etiologies considered. 

After a discussion with vascular surgery colleagues regarding the safety of stopping antiplatelets and/or anticoagulation, the patient’s antiplatelets and anticoagulation were held in order to obtain an LP and possible brain biopsy. His CSF was sent for a broad infectious disease workup, including bacterial and fungal studies. Histoplasma antigens were included in these tests. In the meantime, because of his continued decline, AmBisome® (Gilead Sciences, Inc., La Verne, California, USA) 5 mg/kg daily was started empirically, almost four weeks after his initial presentation. He was scheduled for a brain biopsy; however, his serum Histoplasma antigen ultimately came back positive prior to the operative biopsy. His neurological exam began improving with the initiation of AmBisome®. His LP studies were ultimately unremarkable, including a negative CSF Histoplasma antigen test. The patient was treated with AmBisome® for four weeks and subsequently transitioned to high-dose itraconazole. He continues on itraconazole at this time. His last clinic follow-up was four months after starting treatment. At that time, all of his lesions had reduced or disappeared, and his vasogenic edema had drastically decreased (Figure 1). The largest lesions (left frontal) originally presented at 0.90 cm, with follow-up imaging showing an increase to 1.40 cm, and then a reduction to 1.17 cm after initiation of treatment. The other lesions followed a similar pattern, increasing in size prior to starting treatment and reducing or disappearing after the start of treatment. He was clinically doing well, with no further episodes of weakness or aphasia. He sees infectious diseases monthly at this time.

## Discussion

Screening and diagnostic tests for CNS histoplasmosis

Due to its non-specific clinical presentation and high rate of false negatives, a combination of diagnostic modalities is needed to confirm CNS histoplasmosis. The gold standard for diagnosis of CNS histoplasmosis is a culture of infected CNS tissue or CSF, due to their excellent specificity [10]. CSF culture has high specificity, however, it has low sensitivity, and it may take several weeks to obtain results, potentially delaying the diagnosis and treatment plan. Additionally, testing CSF for anti-Histoplasma IgG and IgM antibody aids antigen detection and improves the sensitivity for the diagnosis of CNS histoplasmosis. *H. capsulatum* polysaccharide antigen (HPA) testing in CSF, urine, or serum has a sensitivity of 78%, 73%, and 50%, respectively [10]. Methods for antibody detection include immunodiffusion (ID), complement fixation (CF), and enzyme immunoassay (EIA) [10,11]. EIA for IgG and IgM anti-Histoplasma antibodies is reportedly more sensitive (82%) than ID (44%), CF (50%), or a combination of ID and CF (51%). EIA shows a specificity of 93% compared to ID and CF combined (96%) [10]. Antibody detection in CSF and serum has a sensitivity of 80-89% and 92%, respectively, although these tests may cross-react to generate false positives [10]. Additionally, positive CSF culture results may resemble those of fungal or TB meningitis infections. To rule out other infectious etiologies, multiple tests must be performed. Up to half of the patients with meningitis due to Histoplasma infection do not present with the typical CSF profile of lymphocytic pleocytosis. In one study, 17% of patients with confirmed CNS histoplasmosis had normal CSF cell count, glucose, and protein. Only 66% of patients had leukocytosis > 5 cells/μL. Protein was >50 mg/mL in 77% of patients, and glucose was <40 mg/mL in 53% [10].

Real-time quantitative polymerase chain reaction (rt-qPCR) assays are also being developed to detect Histoplasma. In one study, the authors demonstrated 100% specificity and 73% sensitivity for 797 clinical specimens [12]. In another study, Alanio et al. demonstrated 97.7% sensitivity in 43 of 44 patients and 99.0% specificity in nine of 863 cases. In culture/microscopy-positive specimens, the positivity rate was 92.2%. Interestingly, RT-qPCR results were also positive in 10/30 culture-negative cases (33.35%), suggesting that making a diagnosis on culture/microscopy alone can generate false negatives [13].

Moreover, RT-qPCR confirmed a histoplasmosis diagnosis in the blood in 92.3% of immunocompromised patients with dissemination [13]. This highly sensitive blood assay could potentially be used in the monitoring treatment of patients with disseminated histoplasmosis. In patients treated with amphotericin B, blood assays demonstrated a twofold decrease in fungal load every day of treatment [13]. Thus, RT-PCR allows for the quantification of fungal load in the blood to help aid in the treatment management of patients with disseminated histoplasmosis. Before recommending RT-qPCR as a standard diagnostic tool, more independent studies evaluating the sensitivity and specificity of RT-qPCR need to be conducted. The goal of RT-qPCR testing is to monitor decreases in fungal load under different therapeutic regimens to show that rapid decreases correlate with better patient outcomes.

In our case, initially, an LP and brain biopsy were avoided in favor of less invasive diagnostics due to the patient’s DAPT therapy and borderline pancytopenia status. Instead, MRS was performed to help determine the etiology of the patient’s multiple CNS brain-enhancing lesions. On MRS, fungal abscesses may contain elevated lipids (1.2-1.3 ppm), lactate (1.3 ppm), alanine (1.5 ppm), acetate (1.9 ppm), succinate (2.4 ppm), choline (3.2 ppm), and trehalose (3.6 ppm), the latter being a distinctive constituent of the fungal cell wall [14]. Vachhani et al. demonstrated mildly decreased N-acetyl aspartate to creatine ratio (1.10 compared with 1.81 on the contralateral side) and mildly elevated choline to creatine ratio (0.90 compared with 0.85) on MRS in patients with intracranial Aspergillus, Candida, mucormycosis, and blastomycosis [9]. Interestingly, a characteristic peak between 3.6 and 3.8 ppm, thought to represent a "trehalose" peak, appears to be specific to fungi and can be useful in differentiating fungal abscesses from malignant neoplasms [9]. Future MRS studies of CNS histoplasmosis need to be conducted to confirm if this prototypical fungal MRS profile is also seen in intracranial Histoplasma infections.

Differential diagnosis

**Figure 3:**
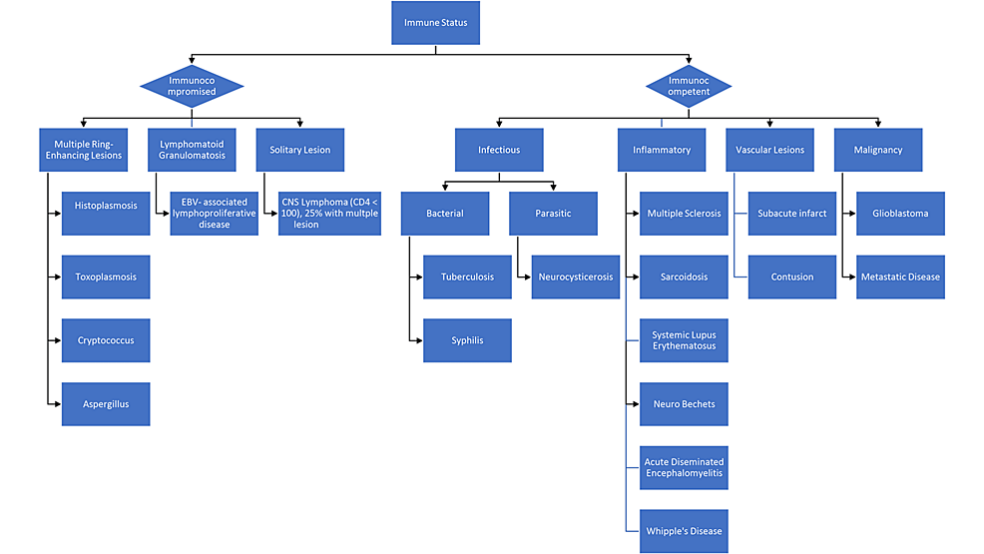
Differential diagnosis for multiple central nervous system ring-enhancing lesions.

The differential diagnosis for multiple CNS ring-enhancing lesions is broad and includes infectious, inflammatory, and malignant etiologies (Figure 3). In an immunocompromised patient, infectious etiologies that should be considered include Cryptococcus, Toxoplasmosis, Aspergillus, and Histoplasmosis. In an immunocompetent patient, tuberculosis, syphilis, and neurocysticercosis should be considered. Primary CNS lymphoma and metastatic disease should also be considered in immunocompromised and immunocompetent patients, respectively. CNS histoplasmosis should be considered when any patient with a history of histoplasmosis, regardless of immunocompetency, presents with ring-enhancing lesions and/or signs and symptoms of meningitis.

Recommended course of treatment

Due to the presentation and difficulty in diagnosis as described, the diagnosis of CNS histoplasmosis is often delayed by more than one month in up to 60% of cases [4,8]. We recommend an empiric four-to six-week course of amphotericin B in any patient with multiple ring-enhancing lesions and a history of prior Histoplasma infection, even in the setting of negative serology. It is important to note that Histoplasma antigen concentrations in the urine and serum fall with effective therapy and should be checked monthly for the first few months and then at three-month intervals while on therapy. It is also imperative to check the following labs at least twice weekly: basic metabolic panel (BMP), calcium (Ca), magnesium (Mag), and phosphorus (PHOS). Also, we recommend checking your weekly CBC with differential and liver function tests. There is no definitive treatment for CNS histoplasmosis. The recommended treatment is liposomal amphotericin B (5 mg/kg daily for a total of 175 mg/kg over four to six weeks) followed by itraconazole (200 mg 2-3 times daily) for at least one year and until resolution of CSF abnormalities, including negative Histoplasma antigen [1]. Our patient was started on this protocol and remains on itraconazole, followed by infectious disease in the outpatient setting with monthly visits.

## Conclusions

This case shows that there is a risk of CNS histoplasmosis even in immunocompetent patients who have been treated for prior infections. In the absence of a clear alternative etiology, we suggest empirically treating any patient with a history of histoplasmosis and multifocal ring-enhancing masses on imaging. Further, empiric treatment with interval imaging follow-up especially should be considered in the setting of small, sub-centimeter lesions in which the yield of an invasive biopsy may be low.
